# Iron, Zinc, Folate, and Vitamin B-12 Status Increased among Women and Children in Yaoundé and Douala, Cameroon, 1 Year after Introducing Fortified Wheat Flour

**DOI:** 10.3945/jn.116.245076

**Published:** 2017-06-07

**Authors:** Reina Engle-Stone, Martin Nankap, Alex O Ndjebayi, Lindsay H Allen, Setareh Shahab-Ferdows, Daniela Hampel, David W Killilea, Marie-Madeleine Gimou, Lisa A Houghton, Avital Friedman, Ann Tarini, Rosemary A Stamm, Kenneth H Brown

**Affiliations:** 1 Department of Nutrition, University of California, Davis, Davis, CA;; 2 Helen Keller International, New York, NY;; 3 USDA, Agricultural Research Service Western Human Nutrition Research Center, Davis, CA;; 4 Nutrition and Metabolism Center, Children’s Hospital Oakland Research Institute, Oakland, CA;; 5 Pasteur Center, Yaoundé, Cameroon;; 6 University of Otago, Dunedin, New Zealand; and; 7 Bill & Melinda Gates Foundation, Seattle, WA

**Keywords:** fortification, effectiveness, iron, folate, vitamin B-12, zinc, breast milk

## Abstract

**Background:** Few data are available on the effectiveness of large-scale food fortification programs.

**Objective:** We assessed the impact of mandatory wheat flour fortification on micronutrient status in Yaoundé and Douala, Cameroon.

**Methods:** We conducted representative surveys 2 y before and 1 y after the introduction of fortified wheat flour. In each survey, 10 households were selected within each of the same 30 clusters (*n* = ∼300 households). Indicators of inflammation, malaria, anemia, and micronutrient status [plasma ferritin, soluble transferrin receptor (sTfR), zinc, folate, and vitamin B-12] were assessed among women aged 15–49 y and children 12–59 mo of age.

**Results:** Wheat flour was consumed in the past 7 d by ≥90% of participants. Postfortification, mean total iron and zinc concentrations of flour samples were 46.2 and 73.6 mg/kg (target added amounts were 60 and 95 mg/kg, respectively). Maternal anemia prevalence was significantly lower postfortification (46.7% compared with 39.1%; adjusted *P* = 0.01), but mean hemoglobin concentrations and child anemia prevalence did not differ. For both women and children postfortification, mean plasma concentrations were greater for ferritin and lower for sTfR after adjustments for potential confounders. Mean plasma zinc concentrations were greater postfortification and the prevalence of low plasma zinc concentration in women after fortification (21%) was lower than before fortification (39%, *P* < 0.001); likewise in children, the prevalence postfortification (28%) was lower than prefortification (47%, *P* < 0.001). Mean plasma total folate concentrations were ∼250% greater postfortification among women (47 compared with 15 nmol/L) and children (56 compared with 20 nmol/L), and the prevalence of low plasma folate values was <1% after fortification in both population subgroups. In a nonrepresentative subset of plasma samples, folic acid was detected in 77% of women (73% of those fasting) and 93% of children. Mean plasma and breast-milk vitamin B-12 concentrations were >50% greater postfortification.

**Conclusion:** Although the pre-post survey design limits causal inference, iron, zinc, folate, and vitamin B-12 status increased among women and children in urban Cameroon after mandatory wheat flour fortification.

## Introduction

Micronutrient deficiencies affect millions of people globally and contribute to increased morbidity and mortality among vulnerable population groups (1). Fortification of industrially processed staple foods is one promising strategy for reducing micronutrient deficiencies and associated health consequences. Yet, despite the widespread introduction of large-scale food fortification programs, few data are available on their effectiveness for improving micronutrient status among population groups at risk of deficiency, such as women of reproductive age and young children. Information on program effectiveness is particularly scant for nutrients such as zinc, vitamin B-12, and, in low-income settings, folate.

In 2009, before the implementation of mandatory large-scale wheat flour fortification in Cameroon, we conducted a national survey to establish the baseline prevalence of micronutrient deficiencies and to collect information on dietary patterns to inform the development of a food fortification program (2, 3). In response to evidence that micronutrient deficiencies were a public health problem, the government of Cameroon instituted a mandatory food fortification program, launched in August 2011. The program includes the addition of vitamin A to refined vegetable oil (40 IU/g as retinyl palmitate) and the addition of iron (60 mg Fe/kg as ferrous fumarate), zinc (95 mg Zn/kg as zinc oxide), folic acid (5.0 mg/kg), and vitamin B-12 (0.04 mg/kg) to wheat flour.

In 2012, we conducted a survey in Yaoundé and Douala, the 2 major metropolitan areas of Cameroon, to evaluate the impact of the fortification program 1 y after the introduction of fortified food products. The objectives of the present analyses were as follows: *1*) to determine the availability of adequately fortified wheat flour in households and markets, *2*) to evaluate the change in indicators of micronutrient status (iron, zinc, folate, and vitamin B-12) from 2009 to 2012 (i.e., 2 y before and 1 y after the introduction of fortified wheat flour), and *3*) to assess whether the consumption of wheat flour postfortification was related to indicators of micronutrient status.

## Methods

### 

#### Study design and sampling.

This analysis included data from 2 cross-sectional, cluster surveys conducted in 2009 and 2012, which were completed in the same season (4, 5). The baseline survey was conducted in September–December 2009 and was representative nationally and at the level of each of 3 survey strata, including 1 stratum representing Yaoundé and Douala (together comprising ∼20% of the total population) (2, 4). Thirty clusters per stratum were selected by using proportionate-to-population size sampling; only results from the urban stratum (Yaoundé and Douala; 15 clusters/city) are included in the present analysis because the evaluation survey was conducted only in Yaoundé and Douala. The evaluation survey was conducted in October–November 2012 and included the same 30 clusters in Yaoundé and Douala that were sampled in the 2009 survey (5). Sampling within each cluster was conducted by identifying a random start point and systematic sampling of adjacent households. The 2 surveys used identical methods to sample households within each cluster, but individual participants likely differed because the random start points were different for each survey.

For both surveys, the target sample size was 10 households (i.e., 10 women and 10 children)/cluster. Data collection took place 2 d after recruitment; thus, 12–15 households/cluster were selected in anticipation of an attrition rate of 15–30%. In addition to the 10 households that participated in the full study, we recruited an additional 2–3 lactating women/cluster to provide breast-milk samples only to obtain the target of 5 milk samples/cluster. The sample size was based on expected change in mean biomarker concentrations after food fortification (effect sizes ranging from 0.2 to 0.5) and assuming a design effect of 2 (although in the urban stratum selected for this pre-post comparison, the estimated design effects were generally <2).

#### Participant eligibility and consent.

Households were eligible to participate if there was ≥1 child 12–59 mo of age and 1 woman of reproductive age (15–49 y) who was the child’s primary caregiver. Children and women were eligible to participate if they had lived in the household for ≥1 mo and did not have reported severe fever, diarrhea with dehydration, or other severe illness at recruitment or between recruitment and data collection (i.e., during the 72 h before data collection). Caregivers were eligible to provide a breast-milk sample if the breastfeeding child was ≥1 mo of age, regardless of whether the lactating woman or breastfeeding child was selected to participate in the full survey.

Women provided informed oral consent for themselves and the child to participate, with permission from the head of household where appropriate. The 2009 survey was approved by the National Ethics Committee of Cameroon and the Institutional Review Board of the University of California, Davis. In 2012, the National Ethics Committee had suspended activity during a period of reorganization; thus, approval was obtained from the Cameroon Ministry of Public Health and the Institutional Review Board of the University of California, Davis.

#### Data collection.

Bilingual (French and English) interviewers administered questionnaires to collect information on household demographic and socioeconomic characteristics. These included housing material, type of toilet, type of fuel for cooking and light, household water source and location, a possessions index, animal ownership, and the education, occupation, and employment status of the index woman and head of household. Information on the consumption of selected “fortifiable” foods, including wheat flour, was collected by using an FFQ, which inquired about different preparations (e.g., wheat flour in bread or beignet) (2). Respondents were asked how many days during the previous 7 d that they (or the index child) had consumed each food and the number of the times they consumed the food on the last day on which the food was consumed. In 2009 only, 24-h dietary recall interviews (with replicates in a subset of ∼10% on a nonconsecutive day) were conducted to quantify total nutrient intakes of women and children. Details of the dietary data collection methods were reported previously (2, 3, 6).

Venous blood (5–7 mL) was collected into trace element–free tubes containing lithium heparin (Sarstedt). International Zinc Nutrition Consultative Group (IZiNCG) recommendations for the collection and processing of samples for plasma zinc measurement were followed (7). Blood collection and storage containers were covered in foil to prevent degradation of light-sensitive analytes. Blood samples were placed immediately in a cooler with ice packs and centrifuged within 2 h to separate plasma (10 min at 2500 × *g* at room temperature). Plasma samples were placed in aliquots within an opaque, portable dead-air box to minimize exposure to light and dust during sample handling.

In 2012 only, an additional 1–2 mL of blood was collected into tubes containing EDTA (Sarstedt) for the measurement of hemoglobin and malaria infection in whole blood. For both surveys, hemoglobin was measured in venous blood by using a portable photometer (Hemocue 201+), except for some children from whom a capillary sample was obtained (estimated to be <10%). In 2009, current or recent malaria infection was determined by measuring plasma histidine-rich protein 2 (HRP2) concentrations (8). In 2012, malaria was assessed in whole blood by using a rapid diagnostic test (SD Bioline Malaria Ag Pf/Pan; Standard Diagnostics Inc.); individuals with positive rapid diagnostic tests results were treated (Coartem; Novartis Pharmaceuticals) and referred to the nearest health clinic.

In both surveys, breast-milk samples were collected by the casual sampling method described previously (9). The mother was asked to feed her child from the fuller breast. After 30 s, the mother transferred the infant to the other breast and manually expressed 5–10 mL of milk from the first breast. Milk-fat concentration was measured in triplicate immediately after collection by using the Creamatocrit method (10), and the remaining milk sample was remixed and aliquots were placed into storage vials wrapped in foil.

Respondents were asked whether they had any wheat flour in their home and, if so, whether they were willing to provide a sample for micronutrient analysis. When available, ∼10 g wheat flour was collected into sterile, plastic containers covered in foil. Because few households (*n* = 25; ∼8%) had wheat flour at home (most consumed wheat flour as purchased bread or beignets), we also obtained flour samples at nearby markets. The field team attempted to locate the vendor indicated by the respondent(s) where possible; otherwise, the nearest available vendor was selected. Samples were obtained by requesting a sample of ∼10 g from a large sack or by purchasing a small sack and retaining a subsample of ∼10 g (after thorough mixing). Aliquots of plasma, breast milk, and wheat flour were stored in a cooler with ice packs until the end of the day, when they were transferred to a freezer (with backup generator) for storage at ≤−20°C until shipping on dry ice for laboratory analyses.

Plasma indicators of inflammation [C-reactive protein (CRP) and α_1_-acid glycoprotein (AGP)] and iron [ferritin and soluble transferrin receptor (sTfR)] status were measured by ELISA (11). Plasma HRP2 was measured by using a commercial kit (Malaria Ag CELISA; Cellabs) (8), and samples with absorbance of ≥0.1 were considered positive. Plasma zinc and wheat flour zinc and iron were measured by inductively coupled plasma atomic emission spectrometry (12). Method performance characteristics, including assay precision and limits of detection and accuracy for select indicators, are presented in **Supplemental Table 1**.

Due to resource constraints, plasma folate and vitamin B-12 concentrations before fortification were initially measured in a randomly selected subset of 50% of households (13). To increase the sample size for the pre-post comparison, the remaining 50% of baseline samples in Yaoundé and Douala were analyzed, in addition to the full set of postfortification samples. Plasma folate and vitamin B-12 were measured by using the Simultrac-SNB RIA vitamin B-12 [^57^Co]/folate [^125^I] (MP Biomedicals) (13). Breast-milk vitamin B-12 concentration was measured by Immulite/Immulite 1000 Vitamin B-12 competitive chemiluminescence enzyme immunoassay (Siemens Healthcare Diagnostic) (14).

To further explore the forms of folate present in plasma, all postfortification samples for which sufficient remaining plasma was available (*n* = 151 women, *n* = 124 children) were analyzed by isotope dilution LC–tandem MS (isotope dilution LC-MS/MS) based on the method of Fazili et al. (15) (Supplemental Table 1). For unmetabolized folic acid (UFA), values below the limit of detection (LOD; 14% overall) were replaced with a small nonzero value calculated as the square root of the LOD (2). For tetrahydrofolate (THF) and 5-formylTHF, values below the LOD (∼96% and ∼81%, respectively) were replaced with 0. MeFox (pyrazino-s-triazine derivative of 4α-hydroxy-5-methylTHF) and 5-methylTHF were detected in all samples. For the analyses of forms of folate, some clusters were not represented due to insufficient remaining plasma samples, so the population sample was not statistically representative. Women with and without sufficient plasma for LC-MS/MS analyses had similar plasma total folate concentrations, as measured by RIA (45.9 compared with 47.8 nmol/L, respectively, among *n* = 287 women with RIA measurements;* P* = 0.99). For children, individuals with sufficient plasma remaining had 13% greater mean plasma folate concentrations compared with children without remaining plasma (59.9 compared with 53.1 nmol/L, respectively, among *n* = 285 children with RIA measurements; *P* = 0.03). Thus, the results for folate metabolites may overestimate the values for the original population sample.

Total folate concentrations measured by the 2 methods were strongly correlated (*r*_s_ = 0.75 for women, *r*_s_ = 0.62 for children; *P* < 0.0001). Among the subset of women with both measures (*n* = 142), the unweighted mean (95% CI) for the RIA values was 45.9 nmol/L (42.7, 49.1 nmol/L) and the mean for LC-MS/MS values was 42.2 nmol/L (38.7, 45.6 nmol/L). The corresponding values for children (*n* = 121) were 59.9 nmol/L (55.2, 64.6 nmol/L) and 52.0 nmol/L (48.4, 55.6 nmol/L), respectively. Thus, we present RIA values for plasma total folate and LC-MS/MS values for folate metabolites.

#### Statistical analysis.

Data were analyzed by using SAS version 9.4 (SAS Institute, Inc.). The SAS survey procedures were used to obtain appropriate variance estimates. Weighting factors were applied to account for the respective population sizes of Yaoundé and Douala and to adjust for nonresponse within each cluster. For each survey, variables relating to socioeconomic status (described above) were combined by using factor analysis to create a continuous score: the eigenvalues were 4.36 (64% of variance) for the national baseline survey and 1.99 (38% of variance) for the postfortification survey in Yaoundé and Douala.

We calculated the frequency of consumption of wheat flour in the past 7 d, excluding pasta, which is not included in the fortification program. Detailed descriptions of the analytical methods for the 24-h dietary recall data have been reported elsewhere (3, 6). We applied a regression equation to estimate absorbable zinc for women (16) and Estimated Average Requirement (EAR) values from the IZiNCG (7) and the Institute of Medicine (IOM) (17). Iron bioavailability was assumed to be 10% in this urban setting, and we estimated vitamin B-12 absorption as described previously (13). The National Cancer Institute method was used to estimate usual nutrient intake distributions (18). The prevalence of inadequate intake was estimated by the EAR cutoff method, with the exception of iron, for which the full probability method was used in combination with estimated physiologic requirements (17). EAR values for folate and vitamin B-12 were from the US IOM (19). We then simulated the effects of wheat flour fortification on the adequacy of micronutrient intakes, as described previously (3), under the assumption that all wheat flour contained added micronutrients at 75% of the target fortification levels (i.e., additional 45 mg Fe/kg, 71.25 mg Zn/kg, 3.75 mg folic acid/kg, and 0.03 mg vitamin B-12/kg; this assumption was based on the measured iron and zinc contents of the flour samples, but is more optimistic because we modeled added micronutrient content, whereas the measured values refer to total iron and zinc).

To report mean ferritin and zinc concentrations and the prevalence of deficiency, concentrations of these indicators were adjusted for inflammation by using regression analysis (20). Briefly, for each biomarker and population group (women and children), a linear regression model was developed to describe the relation between the biomarker and CRP and AGP, including interactions or quadratic terms that were significant. These equations were then used to adjust individual values to concentrations equivalent to those in the absence of inflammation, defined as CRP and AGP <0.12 mg/L and <0.57 g/L for children and <0.16 mg/L and <0.47 g/L for women, respectively (values representing the 10th percentile of individuals in the survey with CRP <5 mg/L and AGP <1 g/L). Body iron stores (BIS) were estimated from sTfR and adjusted ferritin concentrations (21).

Cutoffs for biochemical values were as follows—anemia: hemoglobin <110 g/L (children and pregnant women) and <120 g/L (nonpregnant women); iron deficiency: inflammation-adjusted plasma ferritin concentrations <12 μg/L (children) or <15 μg/L (women), sTfR >8.3 mg/L, or BIS <0 mg/kg; low plasma zinc: <65 μg/dL (morning; children), <57 μg/dL (afternoon; children), <50 μg/dL (pregnant women), <70 μg/dL (morning; fasting women), <66 μg/dL (morning; nonfasting women), and <59 μg/dL (afternoon; women) (7); low plasma folate: <7 or <10 nmol/L (women or children) (22); insufficient vitamin B-12 status: plasma vitamin B-12 <221 pmol/L; and moderate vitamin B-12 deficiency: plasma vitamin B-12 <148 pmol/L(23). Differences in micronutrient status and other indicators by survey were examined by using SAS survey regression procedures, with a binary variable representing survey year. The outcome variables were unadjusted biomarker concentrations or the proportion of low values, and analyses were conducted with and without controlling for potential covariates. Comparisons with *P* < 0.05 were considered significant.

Propensity scores are one method to control for confounding in the relation between fortified food intake and micronutrient status, with the advantage of reducing the number of covariates in the model (24). To control for differences in propensity to consume fortified products frequently, we created propensity scores for the frequent consumption of wheat flour with the use of logistic regression. We defined frequent consumption as ≥14 times/wk, which is approximately the 75th percentile of consumption. Predictors included stratum (city) and variables related to socioeconomic status as described above, with the exception of the possessions index and animal ownership.

For the adjusted analyses, potential covariates included the following: age; residence in Yaoundé or Douala; frequency of wheat flour intake; household socioeconomic status (continuous score); CRP and AGP (both continuous); current or recent malaria; type of toilet (as a proxy for infection risk); propensity to consume wheat flour ≥14 times/wk; time of day of blood collection, time elapsed between previous meal and blood collection, and time from blood collection to centrifugation (zinc only); breastfeeding status (children); pregnancy or lactation; BMI (women); and breast-milk fat concentration (milk vitamin B-12 only). Quadratic terms were evaluated where the relation between the covariate and outcome did not appear linear, and selected interactions with CRP, AGP, and child age were included. All covariates and selected interaction terms were then added to the regression model, and covariates were sequentially removed if they were not significantly associated with the outcome (*P* > 0.05, *P* > 0.1 for interactions). If removal of a covariate changed the regression coefficient of the “survey year” variable by >20% or changed the significance of the *P* value, the covariate was retained in the model. Age was retained in all models. Regression diagnostics (including residuals, leverage, and tolerance) were assessed for the “full” (all covariates) and final models.

Finally, we conducted several plausibility analyses to assess whether any observed change over time was related to the consumption of fortified foods. First, with the use of 2012 data only, we examined the relations between micronutrient status indicators and the frequency of wheat flour consumption by using Spearman correlations (*r*_s_). Second, to better assess whether the relation between wheat flour intake and micronutrient status differed pre- and postfortification, we used regression analysis to model this relation as the interaction between survey year and frequency of consumption of wheat flour in models predicting micronutrient status. This was an exploratory analysis, because we did not base the sample size on that required to detect interactions.

## Results

Recruitment procedures and participant characteristics, including micronutrient status and dietary intake in the 2009 survey, have been reported elsewhere (2, 4, 5). In general, participants in the 2 surveys were similar, but women and children in the postfortification survey were older (29.1 compared with 27.1 y for women, 32.8 compared with 30.3 mo for children; *P* < 0.05) and women had higher mean CRP concentrations postfortification (3.5 compared with 2.7 mg/L; *P* = 0.0002) (**Table 1**).

**TABLE 1:** Characteristics of women and children who participated in the baseline and postfortification surveys in Yaoundé and Douala, Cameroon^1^

	Baseline (2009)		Postfortification (2012)		
	*n*	Value	*n*	Value	*P*
Women					
Age, y	279	27.1 ± 0.4	302	29.1 ± 0.4	0.002
Pregnant, %	214	11	293	16	0.07
Lactating, %	228	26	302	28	0.71
CRP, mg/L	273	2.67 ± 0.22	305	3.52 ± 0.37	0.0002
AGP, g/L	273	0.73 ± 0.01	305	0.72 ± 0.01	0.070
Inflammation, %	273	18	305	22	0.19
Malaria, %	261	7	299	5	0.30
Children					
Age, y	272	30.3 ± 1.0	303	32.9 ± 0.8	0.039
Male sex, %	288	49	308	50	0.86
HAZ	255	−0.68 ± 0.07	300	−0.63 ± 0.07	0.62
Stunted (HAZ <−2), %	255	13.0	300	15.6	0.29
WAZ	255	−0.12 ± 0.05	300	−0.06 ± 0.06	0.47
Underweight (WAZ <−2), %	255	1.4	300	5.7	0.027
WHZ	255	0.36 ± 0.05	300	0.42 ± 0.07	0.46
Wasted (WHZ <−2), %	255	1.0	300	1.4	0.74
Breastfeeding, %	239	5	281	4	0.69
CRP, mg/L	254	4.20 ± 0.33	297	4.49 ± 0.45	0.58
AGP, g/L	254	0.90 ± 0.02	297	0.97 ± 0.02	0.12
Inflammation, %	254	38	297	46	0.10
Malaria, %	234	13	294	8	0.088

1 Values are means ± SEs unless otherwise indicated. Inflammation was defined as CRP >5 mg/L, AGP >1 g/L, or both. Malaria was defined as positive HRP2 antigen in 2009 or by rapid diagnostic test in 2012. AGP, α_1_-acid glycoprotein; CRP, C-reactive protein; HAZ, height-for-age *z* score; HRP2, histidine-rich protein 2; WAZ, weight-for-age *z* score; WHZ, weight-for-height *z* score.

Among wheat flour samples collected (*n* = 38), 76% were considered fortified (≥14 mg Fe/kg and ≥13 mg Zn/kg) and mean iron and zinc contents of fortified samples (57 mg total Fe/kg; 94 mg total Zn/kg) were close to mandated amounts (60 mg added Fe/kg; 95 mg added Zn/kg) (**Table 2**). Only 34% of samples had total Fe ≥60 mg/kg and total Zn ≥95 mg/kg. To assess whether the mineral results might serve as a reasonable proxy for B-vitamin content, we selected a single “fortified” sample (54.9 mg Fe/kg, 93.6 mg Zn/kg) and a single “unfortified” sample (12.0 mg Fe/kg, 6.2 mg Zn/kg) for B-vitamin analysis. The fortified sample contained 2.84 mg folic acid/kg and 0.038 mg vitamin B-12/kg (compared with the targets of 5 mg/kg and 0.04 mg/kg, respectively), whereas the unfortified sample contained 0.11 mg folic acid/kg and <0.001 mg vitamin B-12/kg.

**TABLE 2:** Mineral contents of wheat flour samples collected from households and markets in Yaoundé and Douala, Cameroon, 1 y after mandatory wheat flour fortification^1^

	Yaoundé	Douala	Total
Iron, mg/kg			
Household	46.4 ± 33.1	49.3 ± 31.8	48.1 ± 31.7
Market	42.6 ± 29.5	43.2 ± 31.2	42.9 ± 29.2
Total	44.8 ± 30.8	47.3 ± 31.0	46.2 ± 30.5
Zinc, mg/kg			
Household	75.6 ± 36.7	72.7 ± 41.4	73.9 ± 38.7
Market	70.2 ± 55.3	76.0 ± 50.5	73.1 ± 51.0
Total	73.4 ± 43.7	73.8 ± 43.4	73.6 ± 43.0

1 Values are unweighted means ± SDs; *n* = 38 wheat flour samples (*n* = 10 households in Yaoundé, *n* = 14 households in Douala, *n* = 7 markets in Yaoundé, *n* = 7 markets in Douala). Overall median values were 40.8 mg Fe/kg and 83.5 mg Zn/kg. Mandated fortification levels are 60 mg Fe/kg and 95 mg Zn/kg.

More than 90% of respondents reported consuming wheat flour in the past week, with an average frequency of 1–2 times/d (**Table 3**). The median and 75th percentile of usual flour intakes among all nonbreastfeeding children in this region (>90% of children aged 12–59 mo) were 31 and 66 g/d, respectively. For women, the median and 75th percentile of usual flour intakes were 24 and 79 g/d (estimates included wheat flour consumers and “nonconsumers”). Dietary modeling predicted that wheat flour fortification at 75% of the target levels would result in large decreases in the prevalence of inadequate folate intakes and more moderate decreases in the prevalence of inadequate iron and vitamin B-12 intakes. For women, the predicted effect of zinc fortification varied according to the EAR [e.g., IZiNCG (7) and IOM (17)] and the assumptions about absorption, and decreased in all cases (Table 3). Among nonbreastfeeding children, the mean usual total zinc intake was predicted to increase from 3.6 to 7.5 mg/d, but with no change in the prevalence of intakes below the EAR (16% with total zinc intake <2 mg/d).

**TABLE 3:** Consumption of fortified foods, prevalence of inadequate micronutrient intakes, and predicted impact of fortification on dietary adequacy among women and children in Yaoundé and Douala, Cameroon^1^

	Women		Children	
	Baseline	Postfortification	Baseline	Postfortification
*n* (FFQ)	290	309	290	309
Wheat flour consumption in past week, %	97.6 ± 0.9	95.2 ± 1.4	97.0 ± 1.0	98.4 ± 0.7
Frequency of wheat flour consumption in past week among consumers, times/wk	10.6 ± 0.4	8.9 ± 0.3^2^	15.2 ± 0.7	12.6 ± 0.5^2^
Frequency of wheat flour consumption (all participants), times/wk	10.3 ± 0.4	8.5 ± 0.3^2^	14.8 ± 0.6	12.4 ± 0.5^2^
*n* (24-h dietary recalls)	297	—	229	—
Mean wheat flour consumption on previous day among consumers,^3^ g/d	96 ± 5	—	77 ± 3	—
Mean usual wheat flour consumption,^3^ g/d	46 ± 2	—	38 ± 1	—
Inadequate iron intake,^4^ %	85	66	60	36
Zinc intake less than the EAR, %	40 ± 3	13 ± 1	16 ± 2	16 ± 1
Absorbable zinc intake, %				
<1.86 mg/d (IZiNCG)	21 ± 1	11 ± 1	—	—
<3.30 mg/d (IOM)	99 ± <1	73 ± 2	—	—
Folate intake (DFEs) less than the EAR, %	79 ± 4	13 ± 1	44 ± 3	16 ± 1
Vitamin B-12 intake less than the EAR, %	22 ± 3	17 ± 2	27 ± 3	19 ± 2
Adjusted vitamin B-12 intake less than the EAR, %	51 ± 2	29 ± 2	31 ± 3	18 ± 2

1 Values are means ± SEs unless otherwise indicated. Simulations assume wheat flour fortification at 75% of the target value (45 mg Fe/kg, 71.3 mg Zn/kg, 3.75 mg folic acid/kg, and 0.03 mg vitamin B-12/kg). Estimates of dietary adequacy are presented only among nonbreastfed children because breast-milk intake was not quantified. The prevalence of inadequate intakes was estimated by using the EAR cutoff method, except for iron, for which the probability method was used, assuming 10% absorption (17). The EAR for total zinc was 6 mg/d for women (moderate bioavailability) and 2 mg/d for children (low-to-moderate bioavailability) (7); phytate-to-zinc molar ratios were 12–14. Absorbable zinc was estimated for women according to Miller et al. (16); EAR values from the IZiNCG (7) and the IOM (17) were applied. Folic acid was converted to DFEs by multiplying by 1.67. Adjusted vitamin B-12 intake was calculated as follows: for each serving of food that included >3 μg vitamin B-12, the amount of vitamin B-12 was divided by 5 to adjust for lower absorption from higher doses (13). EAR values for folate were 320 μg DFEs/d for women and 120 μg DFEs/d for children; EAR values for vitamin B-12 were 2.0 μg/d for women and 0.7 μg/d for children (19). DFE, Dietary Folate Equivalent; EAR, Estimated Average Requirement; IOM, Institute of Medicine; IZiNCG, International Zinc Nutrition Consultative Group.

2 Different from the prefortification value, *P* < 0.05.

3 Mean wheat flour consumption on the previous day among consumers estimated without adjustment for intraindividual variation. Mean usual wheat flour consumption was estimated by the National Cancer Institute method, as described in the text. Estimates for children excluded breastfeeding children.

4 Inadequate iron intake was calculated by applying the probability method to the usual intake distribution, assuming 10% absorption; SEs were not generated for this method.

In Yaoundé and Douala before fortification, almost half of women and children were anemic, and deficiencies in iron and zinc were common (**Tables 4** and **5**). Low plasma folate and vitamin B-12 concentrations were less common but still present, particularly for folate among women.

**TABLE 4:** Hemoglobin concentrations, plasma concentrations of micronutrient status indicators, and breast-milk vitamin B-12 concentrations among women in Yaoundé and Douala, Cameroon, 2 y before and 1 y after the introduction of fortified wheat flour^1^

			*P*	
	Baseline (2009)	Postfortification (2012)	Unadjusted^2^	Adjusted^3^
*n*	285	307		
Hemoglobin, g/L	119 ± 1	120 ± 1	0.36	0.078
Anemia, %	46.7 ± 2.7	39.1 ± 2.7	0.061	0.013
*n*	273	305		
Ferritin, μg/L	37.0 ± 1.5	47.3 ± 2.0	0.010	0.046
Adjusted^4^ ferritin, μg/L	30.0 ± 1.1	37.6 ± 1.6	0.019	—
Ferritin <15 μg/L, %	19.6 ± 3.1	14.4 ± 2.2	0.20	0.41
Adjusted^4^ ferritin <15 μg/L, %	24.3 ± 3.3	19.3 ± 2.4	0.23	—
sTfR, mg/L	7.73 ± 0.18	6.23 ± 0.17	<0.0001	<0.0001
sTfR >8.3 mg/L, %	30.6 ± 3.0	8.6 ± 1.6	<0.0001	<0.0001
BIS, mg/kg	3.55 ± 0.21	5.04 ± 0.17	<0.0001	0.0006
Adjusted^4^ BIS, mg/kg	2.88 ± 0.20	4.31 ± 0.18	0.0002	—
BIS <0 mg/kg, %	14.6 ± 2.2	7.5 ± 1.2	0.004	0.041
Adjusted^4^ BIS <0 mg/kg, %	17.8 ± 2.5	11.5 ± 1.9	0.0095	—
Iron deficiency and anemia,^5^ %	18.0 ± 2.7	13.1 ± 1.8	0.17	0.30
*n*	284	290		
Plasma zinc, μg/dL	55.1 ± 0.6	65.2 ± 1.5	<0.0001	<0.0001
Low plasma zinc,^6^ %	75.0 ± 2.4	52.0 ± 4.0	<0.0001	<0.0001
*n*	271	290		
Adjusted^4^ plasma zinc, μg/dL	64.5 ± 0.8	79.5 ± 2.0	<0.0001	—
Low adjusted^4^ plasma zinc,^4^^,^^6^ %	39.4 ± 2.6	21.6 ± 3.3	<0.0001	—
*n*	195	287		
Plasma folate, nmol/L	14.8 ± 0.7	46.9 ± 1.2	<0.0001	<0.0001
<7 nmol/L, %	6.1 ± 1.5	0.3 ± 0.3	<0.0001	<0.0001
<10 nmol/L, %	30.1 ± 4.3	0.3 ± 0.3	<0.0001	<0.0001
>45 nmol/L, %	0.6 ± 0.6	46.1 ± 4.2	<0.0001	<0.0001
Plasma vitamin B-12, pmol/L	461 ± 18	671 ± 24	<0.0001	<0.0001
<221 pmol/L, %	12.8 ± 3.1	3.8 ± 1.3	0.0032	0.002
<148 pmol/L, %	8.0 ± 2.5	1.8 ± 0.9	0.011	0.025
*n*	23	133		
Breast-milk vitamin B-12, pmol/L	333 ± 46	685 ± 31	<0.0001	0.0004
<221 pmol/L, %	37.2 ± 12.0	7.2 ± 2.7	0.0004	0.001

1 Values are means ± SEs unless otherwise indicated. Sample size differs by analytical method. AGP, α_1_-acid glycoprotein; BIS, body iron stores; CRP, C-reactive protein; sTfR, soluble transferrin receptor.

2 Without covariates, unless otherwise noted.

3 Controlling for covariates, as described in Methods.

4 Adjusted for inflammation by regression analysis to values equivalent to those at CRP and AGP concentrations of 0.12 mg CRP/L and 0.57 g AGP/L for children and 0.16 mg CRP/L and 0.47 g AGP/L for women (10th percentile among individuals with CRP <5 mg/L and AGP <1 g/L). Values for the 2 surveys were compared by regression analysis with the use of the unadjusted value as the dependent variable and including CRP, AGP, and their interaction as covariates.

5 Iron deficiency and anemia were defined as inflammation-adjusted ferritin <15 μg/L and hemoglobin <110 g/L (pregnant women) or <120 g/L (nonpregnant women). Values for the 2 surveys were compared by using “unadjusted iron deficiency and anemia” as the dependent variable, with and without including CRP, AGP, their interaction, and other covariates. The prevalence of inflammation-adjusted iron deficiency and anemia did not differ by survey (*P* = 0.08).

6 Low plasma zinc was defined as <50 μg/dL for pregnancy, <70 μg/dL for morning fasting samples, <66 μg/dL for morning nonfasting samples, and <59 μg/dL for afternoon samples (7). To compare the prevalence of low plasma zinc, a single cutoff of 66 μg/dL was used, and time of day of blood collection and time elapsed between blood collection and the previous meal were included as covariates.

**TABLE 5:** Hemoglobin and plasma concentrations of micronutrient status indicators among children in Yaoundé and Douala, Cameroon, 2 y before and 1 y after the introduction of fortified wheat flour^1^

			*P*	
	Baseline (2009)	Postfortification (2012)	Unadjusted^2^	Adjusted^3^
*n*	276	302		
Hemoglobin, g/L	111 ± 1	110 ± 1	0.30	0.20
Anemia, %	46.7 ± 2.9	45.5 ± 3.0	0.73	0.95
*n*	254	297		
Ferritin, μg/L	38.6 ± 2.2	51.1 ± 2.8	0.0003	0.0001
Adjusted^4^ ferritin, μg/L	23.5 ± 1.3	30.8 ± 1.1	<0.0001	—
Ferritin <12 μg/L, %	13.1 ± 2.8	6.7 ± 1.5	0.027	0.009
Adjusted^4^ ferritin <12 μg/L, %	27.7 ± 3.5	15.5 ± 2.1	0.021	—
sTfR, mg/L	10.58 ± 0.30	8.22 ± 0.20	<0.0001	<0.0001
sTfR >8.3 mg/L, %	63.9 ± 3.7	25.4 ± 2.3	<0.0001	<0.0001
BIS, mg/kg	2.49 ± 0.24	4.34 ± 0.17	<0.0001	<0.0001
Adjusted^4^ BIS, mg/kg	0.86 ± 0.23	2.70 ± 0.15	<0.0001	—
BIS <0 mg/kg, %	22.2 ± 3.5	8.1 ± 1.6	0.0001	<0.0001
Adjusted^4^ BIS <0 mg/kg, %	35.8 ± 3.9	16.2 ± 2.1	0.0001	—
Iron deficiency and anemia,^5^ %	16.4 ± 2.6	9.9 ± 1.8	0.22	0.21
*n*	265	290		
Plasma zinc, μg/dL	56.6 ± 0.9	66.7 ± 1.6	<0.0001	0.002
Low plasma zinc,^6^ %	60.8 ± 3.5	43.1 ± 4.4	0.0007	<0.0001
*n*	249	288		
Adjusted^4^ zinc, μg/dL	61.0 ± 0.8	72.2 ± 1.7	<0.0001	—
Low adjusted^4^ plasma zinc,^6^ %	46.8 ± 3.9	28.4 ± 4.2	0.0003	—
*n*	183	285	<0.0001	<0.0001
Folate, nmol/L	19.9 ± 0.9	56.0 ± 2.1	<0.0001	<0.0001
<7 nmol/L, %	2.0 ± 1.0	0	<0.0001	<0.0001
<10 nmol/L, %	12.5 ± 2.4	0	<0.0001	<0.0001
>45 nmol/L, %	3.0 ± 1.3	68.8 ± 4.2	<0.0001	<0.0001
Plasma vitamin B-12, pmol/L	466 ± 19	816 ± 39	<0.0001	<0.0001
<221 pmol/L, %	11.9 ± 2.4	1.8 ± 0.9	0.0008	<0.0001
<148 pmol/L, %	6.3 ± 1.4	1.4 ± 0.7	0.0023	0.002

1 Values are means ± SEs unless otherwise indicated. AGP, α_1_-acid glycoprotein; BIS, body iron stores; CRP, C-reactive protein; sTfR, soluble transferrin receptor.

2 Without covariates, unless otherwise noted.

3 Controlling for covariates, as described in Methods.

4 Adjusted for inflammation by regression analysis to values equivalent to those at CRP and AGP concentrations of 0.12 mg CRP/L and 0.57 g AGP/L for children and 0.16 mg CRP/L and 0.47 g AGP/L for women (10th percentile among individuals with CRP <5 mg/L and AGP <1 g/L). Values for the 2 surveys were compared by regression analysis with the use of the unadjusted value as the dependent variable and including CRP, AGP, and their interaction as covariates.

5 Iron deficiency and anemia were defined as inflammation-adjusted ferritin <12 μg/L and hemoglobin <110 g/L. Values for the 2 surveys were compared by using “unadjusted iron deficiency and anemia” as the dependent variable, with and without including CRP, AGP, their interaction, and other covariates. The prevalence of inflammation-adjusted iron deficiency and anemia differed by survey (*P* = 0.036).

6 Low plasma zinc was defined as <65 μg/dL for morning samples and <57 μg/dL for afternoon samples (7). To compare the prevalence of low plasma zinc, a single cutoff of <65 μg/dL was used, and time of day of blood collection was included as a covariate.

Postfortification, mean maternal hemoglobin concentrations did not differ from prefortification values (adjusted *P* = 0.078); however, the prevalence of anemia was significantly lower postfortification (39.1% compared with 46.7%; *P* = 0.06, unadjusted; *P* = 0.01, adjusted for age, pregnancy, malaria, and inflammatory markers; Table 4). There were no differences by survey year in hemoglobin concentration or anemia prevalence among children, with or without controlling for covariates (Table 5).

Among women, mean estimated BIS and plasma concentrations of ferritin were greater and sTfR concentrations were lower after fortification, with and without controlling for age, pregnancy, malaria, and inflammation. Postfortification, the prevalence of iron deficiency, as measured by BIS and sTfR, was lower, but the prevalences of low plasma ferritin and iron deficiency anemia (defined as low inflammation-adjusted plasma ferritin concentrations and anemia) did not differ. Among children, all indicators suggested increased iron status, with and without controlling for inflammation, malaria, age, and other potential confounders, but the prevalence of iron deficiency anemia did not differ between surveys.

Compared with 2009, mean plasma zinc concentrations in 2012 were greater and the prevalence of low values was lower for both women and children. These differences persisted after adjusting for age, breastfeeding status (children), pregnancy (women), malaria, inflammation, household characteristics, and variables related to the timing of blood sampling.

Mean plasma folate concentrations were almost 300% greater among women (47 compared with 15 nmol/L) and children (56 compared with 20 nmol/L) in 2012 compared with those in 2009, and the prevalence of low plasma folate values was reduced to <1% (Tables 4 and 5). Similarly, mean plasma vitamin B-12 concentrations were >50% greater among both women and children in 2012 compared with 2009, and mean breast-milk vitamin B-12 concentrations doubled (Table 4). These differences were significant with and without adjusting for covariates.

Among postfortification samples with sufficient remaining plasma for the measurement of folate forms, 5-methylTHF and MeFox contributed the largest proportion to total folate concentrations (**Table 6**). UFA was detected (≥0.44 nmol/L) in plasma among 77% of women and 93% of children (**Supplemental Table 2**, Table 6). The proportions of UFA relative to total folate concentrations were 7.0% among children and 3.3% among women. However, nonfasting status has been associated with elevated concentrations of most forms of folate (i.e., reflecting the transient effects of recent intake), and only 11% of the blood samples from the children were collected after ≥8 h of fasting (by caregiver report). Stratification of women by fasting status (≥8 h) showed lower UFA in the fasting state (mean: 0.8 nmol/L among 41% of women) than in the nonfasting state (1.9 nmol/L; *P* < 0.05), although plasma total folate did not differ by fasting status. Among fasting women, the proportion of UFA relative to total folate was 2.1% on average. Overall, UFA concentrations were positively associated with higher plasma total folate as measured by LC-MS/MS (*r*_s_ = 0.50 for women and *r*_s_ = 0.51 for children, *P* < 0.001).

**TABLE 6:** Plasma concentrations of folate metabolites among women (fasting and nonfasting) and children 1 y after the introduction of fortification of wheat flour with folic acid^1^

	Women (all)	Women (fasting)	Women (nonfasting)	Children (all)
*n*	151	59	90	124
5-MethylTHF, nmol/L	31.0 (24.9, 40.9)	31.3 (26.1, 42.1)	30.7 (24.0, 39.5)	38.6 (29.5, 47.8)
Non-methyl folate, nmol/L	0 (0, 0)	0 (0, 0)	0 (0, 0)	0 (0, 0.80)
Unmetabolized folic acid, nmol/L	0.85 (0.56, 1.42)	0.69 (0.48, 1.01)	1.02 (0.70, 1.71)	1.51 (0.77, 4.64)
MeFox, nmol/L	7.2 (5.4, 9.2)	6.6 (5.1, 8.9)	7.4 (5.8, 9.4)	8.4 (7.0, 10.6)
Total (calculated), nmol/L	41.2 (32.3, 50.4)	39.7 (33.5, 50.2)	41.4 (31.7, 50.4)	52.4 (39.6, 64.0)
Folic acid, %				
More than the LOD	77	73	79	93
>1 nmol/L	41	25	51	66
>4 nmol/L	4.0	0	6.7	28.2

1 Values are medians (25th, 75th percentiles) unless otherwise indicated. “Fasting” was defined as reported not consuming foods or beverages for ≥8 h before blood collection. Fasting results are not presented for children because only 11% of children were reported as fasting at the time of blood collection. Non-methyl folate represents the sum of 2 minor forms: tetrahydrofolate and 5-formyltetrahydrofolate. Total folate is the sum of all folate forms including MeFox. Among 15 children who were reported by the caregiver to be fasting at the time of blood collection, 7 had folic acid concentrations >1 nmol/L and 1 had a folic acid concentration >4 nmol/L. LOD, limit of detection (0.44 nmol/L for folic acid); MeFox, pyrazino-s-triazine derivative of 4α-hydroxy-5-methylTHF; 5-MethylTHF, 5-methyltetrahydrofolate.

In 2012, the frequency of flour consumption was positively correlated with plasma folate (*r*_s_ = 0.19, *P* = 0.001) and vitamin B-12 (*r*_s_ = 0.12, *P* = 0.052) among women and with plasma vitamin B-12 concentration among children (*r*_s_ = 0.19, *P* = 0.002). UFA concentrations were not correlated with flour intake frequency (*r*_s_ = 0.08, *P* = 0.31, *n* = 151 for women; *r*_s_ = 0.11, *P* = 0.23, *n* = 123 for children). Correlations between flour intake frequency and indicators of iron and zinc status were not significant among women or children. The frequency of flour consumption was positively correlated with hemoglobin among children (*r*_s_ = 0.12, *P* = 0.04) but not among women (*r*_s_ = 0.02, *P* = 0.70).

**FIGURE 1:**
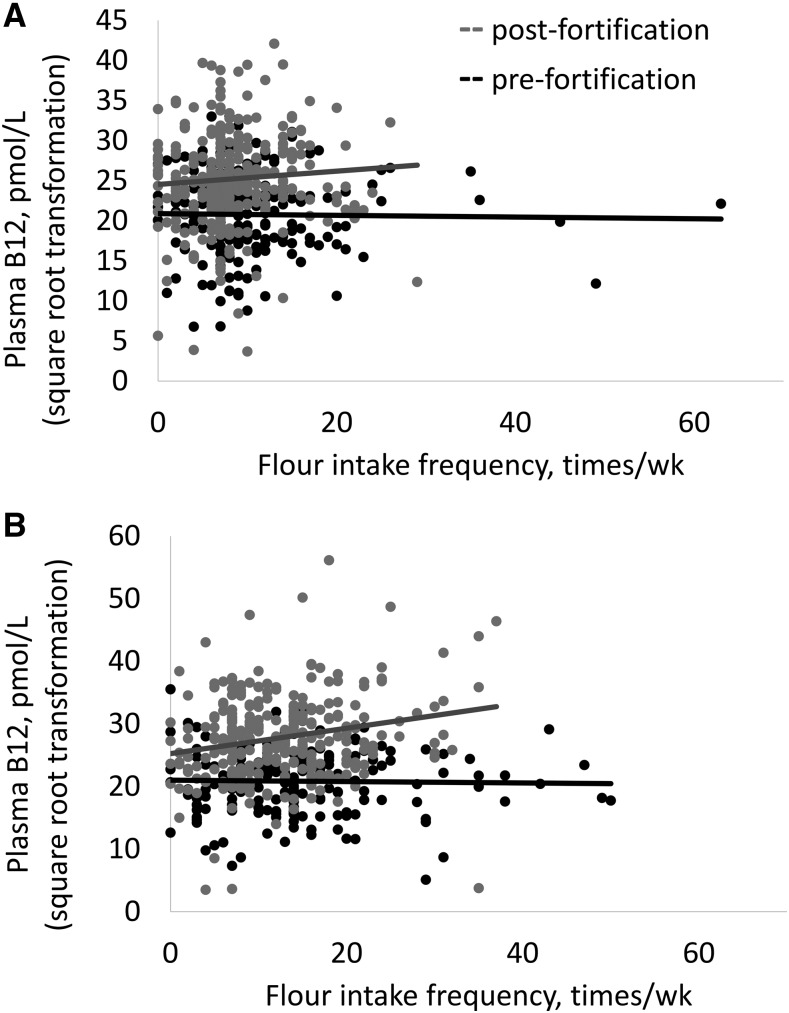
Relations between plasma vitamin B-12 concentrations (after square root transformation) among women (A) and children (B) and the frequency of wheat flour intake in the past week, pre- and postfortification. For women, adjusted *P* = 0.025 for the interaction, controlling for pregnancy, lactation, strata, age, and AGP (*n* = 465); for children, adjusted *P* = 0.06 for the interaction, controlling for age, height-for-age *z* score, plasma AGP, breastfeeding status, and household waste disposal facility (*n* = 395). Truncating flour frequency values to 30 times/wk resulted in *P* values of 0.059 for women and 0.027 for children. AGP, α_1_-acid glycoprotein; B12, vitamin B-12.

In linear regression models, there was a significant interaction between survey year and the frequency of flour intake in predicting plasma vitamin B-12 concentrations in women (*P* = 0.025) and a marginal interaction in children (*P* = 0.063). Specifically, in 2009, there was no relation between wheat flour consumption and plasma vitamin B-12, but in 2012 there was a positive relation between these variables (**Figure 1**). In addition, there was a significant interaction for breast-milk vitamin B-12 concentrations (*P* = 0.03) in which there was no relation between flour intake frequency and breast-milk vitamin B-12 at baseline (*n* = 23) and a negative relation between breast-milk vitamin B-12 and flour intake in 2012 (*n* = 113). For all other micronutrient indicators, interactions between survey year and the frequency of wheat flour intake were not significant.

## Discussion

Before fortification, multiple micronutrient deficiencies were present among both women and children in this urban setting. Wheat flour consumption was common and frequent. One year after the introduction of fortified wheat flour, 76% of wheat flour samples collected from households and markets were designated as fortified, and the mean amounts of minerals in fortified samples were close to the mandated amounts. Nutritional status for all micronutrients added to the wheat flour was greater among both women and children postfortification. Particularly large differences were observed in indicators of folate and vitamin B-12 status. The positive relation between flour consumption and vitamin B-12 biomarkers postfortification, but not prefortification, suggested that the change in status was related to the consumption of fortified flour, although this relation was not observed for other nutrients. In addition, hemoglobin concentrations were slightly greater postfortification among women, but not children, after controlling for malaria, inflammation, and other covariates.

Although unrelated to the primary objectives of this analysis, we observed a significantly greater prevalence of underweight, but not wasting, among children in the postfortification survey, which remained significant after controlling for child age. The Demographic and Health Survey for Cameroon reported a similar prevalence of underweight among children aged 6–59 mo in Yaoundé and Douala in 2004 (3.8%) and 2011 (3.4%) (25), so our finding may be an anomaly, although this should be confirmed.

In the national baseline survey, we observed regional differences in micronutrient deficiency prevalence and consumption of fortifiable foods, which have implications for program effectiveness (2). Micronutrient deficiencies were generally more common in the South and North strata than in Yaoundé and Douala, with the exception of folate intake and status, which were lowest in Yaoundé and Douala (2, 13). Wheat flour products were consumed in greater amounts in Yaoundé and Douala than in the 2 other strata (2). The wheat flour fortification levels in Cameroon correspond to a mean wheat flour intake of <75 g/d according to WHO recommendations (26), which was consistent with flour consumption nationally among consumers and nonconsumers combined. However, the mean flour consumption among women who consumed flour on the previous day, particularly in Yaoundé and Douala, was in the range of 75–149 g/d, which would correspond to lower fortification levels for all nutrients except for iron. Thus, the program would be expected to contribute the most additional micronutrients to diets in the urban areas, but the impact on deficiency prevalence is potentially greater in other regions of Cameroon.

Several limitations of this study relate to data availability. Resources did not permit the collection of 24-h dietary recall data in 2012 to quantify the contribution of fortification to micronutrient intakes. Flour samples were collected at a single time point, preventing detection of fluctuations in average fortification levels over time, and resources did not permit the measurement of folate and vitamin B-12 in all flour samples. Because we aimed to evaluate a national program, it was not possible to include a control group. The pre-post design limits our ability to attribute changes in micronutrient status to the fortification program, because it is possible that unmeasured factors contributed to the observed differences. However, the biochemical measures were conducted in the same laboratories with the use of the same methods (with the exception of malaria), minimizing interlaboratory variation. Statistically controlling for important potential confounders did not explain the greater micronutrient status in 2012 than in 2009.

Postfortification, wheat flour intake was positively associated with maternal plasma folate and with plasma vitamin B-12 among women and children. However, such a relation is insufficient to assess whether changes in micronutrient status are attributable to a fortification program because we previously observed correlations between micronutrient biomarkers and fortifiable food intake before fortification, likely due to underlying factors such as socioeconomic status (2). The availability of baseline data permitted examination of potential changes in the relation between fortified food intake and micronutrient status postfortification, although we had limited statistical power to detect interactions and the measure of dietary intake available at both time points (frequency of consumption of selected foods) limited the scope and precision of plausibility analyses. Nevertheless, the observed relations between plasma vitamin B-12 and flour intake in different survey years are consistent with a dose-response relation between flour intake and vitamin B-12 status that appeared only after fortification. The interaction between milk vitamin B-12 and flour consumption appears to be inconsistent but is difficult to interpret due to the small baseline sample size.

A number of studies have documented decreases in the prevalence of anemia and iron deficiency among women and children after fortification with appropriate forms of iron (27). In contrast, there is less evidence for the effectiveness of zinc fortification. A recent review concluded that zinc added alone to foods may increase serum zinc, but zinc added with other micronutrients may have no effect on serum zinc (28). In controlled efficacy studies, plasma zinc did not respond to the consumption of zinc-fortified foods after 6 mo among children 6–8 mo of age in Peru (3 mg Zn/d) (29) and after 4 wk among men ≥18 y of age in Senegal (7.5 or 15 mg Zn/d) (30). However, serum zinc concentrations were greater after 24 and 36 mo among women in rural China whose families received wheat flour fortified with zinc and other nutrients than in women who received unfortified wheat flour (31). It is possible that the longer durations of the study in China and the present study contributed to the difference in results.

The apparent large increases in plasma folate concentrations are similar in magnitude to those observed in other settings in which folic acid fortification was implemented. For example, in the United States, serum folate concentrations among women increased >2-fold and the prevalence of low serum folate decreased from 21% to <1% between the NHANES 1988–1994 (before fortification) and the NHANES 2003–2004 (32); the fortification level was lower (1.4 μg/g) but enriched grain intakes were presumably higher. Furthermore, mean serum total folate as measured by LC-MS/MS among supplement nonuser children 1–5 y of age (51.3 nmol/L) from the NHANES 2007–2008 survey period was comparable to that among children (52.9 nmol/L) in the present study. Moreover, UFA was detected in nearly all US serum samples from NHANES 2007–2008, regardless of fasting status (33); the authors suggested that higher concentrations of UFA in circulation may indicate that dietary intake exceeds requirements. Thus, the higher UFA concentrations observed in the current study may indicate that the folic acid fortification level is higher than necessary for these cities. The effects in other regions of Cameroon, where flour intake was lower, are unknown; these data are urgently needed to inform adjustments to the program at the national level.

Few countries have large-scale vitamin B-12 fortification programs, and to our knowledge, this is the first evaluation of the effectiveness of large-scale flour fortification on vitamin B-12 status. The results suggest that vitamin B-12 fortification may have a large impact on vitamin B-12 status where wheat flour is frequently consumed. Furthermore, the greater milk vitamin B-12 concentrations that we observed postfortification surpassed the effects observed in supplementation studies (34), which could be related to greater vitamin B-12 absorption from small frequent doses than from larger, less frequent supplemental doses.

In summary, we observed greater iron, zinc, folate, and vitamin B-12 status and a lower prevalence of deficiencies of these micronutrients among women of reproductive age and children aged 12–59 mo in urban Cameroon, and a slightly lower prevalence of anemia among women, 1 y after the introduction of mandatory wheat flour fortification with micronutrients. Although the pre-post survey design limits inferences about causality, the available data suggest that the observed changes can be attributed to the fortification program. Program managers interested in using a similar approach to track changes in micronutrient status over time may wish to forego the complexity of statistical plausibility analyses, but would need to invest similar effort in the careful measurement of biomarkers to ensure that investments result in programs that are safe and effective. Although young children are often assumed to consume insufficient quantities of staple foods to be affected by large-scale fortification, our results suggest that young children should not be ignored as potential beneficiaries [as observed elsewhere (35)], although the programs are unlikely to fully meet the needs for all nutrients, particularly minerals. The apparent lack of effect on child anemia prevalence suggests that other interventions to address anemia among children may be needed. Analysis of wheat flour samples indicated that only ∼75% of samples were fortified, indicating the need to strengthen efforts to monitor the quality of fortified products and to enforce the norms adopted by the government. The high plasma folate and UFA concentrations suggest that the current fortification levels are greater than needed in these cities. The program should be evaluated in other regions of Cameroon where deficiencies in most micronutrients (and thus potential to benefit from fortification) were greatest, to inform adjustments to program design at the national level.
